# Association Between Healthy Lifestyle Habits and Intrinsic Capacity Among Community-Dwelling Older Adults in Singapore

**DOI:** 10.3390/nu18060918

**Published:** 2026-03-14

**Authors:** Jeremy Teng Jun Wei, Shuna S. Khoo, Reshma A. Merchant, Li Feng Tan, Lile Jia, on behalf of the Health District @ Queenstown

**Affiliations:** 1 Division of Geriatric Medicine, Alexandra Hospital, 378 Alexandra Road, Singapore 159964, Singapore; 2 Department of Psychology, Faculty of Arts and Social Sciences, National University of Singapore, 9 Arts Link, Singapore 117570, Singapore; 3 Division of Geriatric Medicine, National University Hospital, 1E Kent Ridge Road, Singapore 119228, Singapore; 4 Yong Loo Lin School of Medicine, National University of Singapore, 10 Medical Drive, Singapore 117597, Singapore

**Keywords:** intrinsic capacity, healthy lifestyle, healthy ageing, population health

## Abstract

Background: Intrinsic capacity (IC) is the composite of an individual’s physical and mental capacities. While lifestyle factors influence health outcomes, their combined association with IC remains understudied. Objective: To examine the association between a Healthy Lifestyle Score (HLS) and intrinsic capacity in older adults in Singapore. Methods: Data from a population-based sample of older adults aged ≥60 years in the Queenstown district of Singapore was analysed. The HLS (range 0–5) included smoking, alcohol use, physical activity, sleep quality, and BMI (Asian cut-offs). IC was measured using the WHO ICOPE framework and defined as the presence of one or more deficits. Results: A total of 1644 participants were included (mean age 72.1 years, 56.4% women). IC deficits were present in 50.9% of the cohort. Based on HLS, 29.9% were classified as unhealthy (0–2), 41.4% intermediate (3), and 28.6% healthy (4–5). HLS category was significantly associated with IC deficits (*p* = 0.004). Among participants with healthy lifestyles, 55.6% had no IC deficits, compared to 47.0% in the intermediate and 45.9% in the unhealthy groups. Only 13.9% met recommended physical activity levels; 58.3% had an unhealthy BMI, 20.0% consumed alcohol, 8.1% were smokers, and 31.7% reported insufficient sleep. Conclusions: Healthier lifestyle profiles are significantly associated with fewer IC deficits. These findings underscore the importance of promoting modifiable health behaviours to preserve intrinsic capacity and support healthy ageing.

## 1. Introduction

With the advancement of technology, public health measures and improvements in healthcare delivery worldwide, global life expectancy has increased markedly over the past century [1]. However, gains in longevity have not been matched by proportional improvements in healthspan, defined as the period of life spent in good health and functional independence [1]. While people are living longer, a growing proportion of these additional years are lived with chronic disease, disability, and functional impairment, resulting in an expanding burden of morbidity at both individual and societal levels [2,3]. Population ageing has therefore been accompanied by rising prevalence of multimorbidity, frailty, and functional dependence, contributing to increased healthcare utilisation, long-term care needs, caregiver burden, and escalating healthcare costs [2,4].

In response to these challenges, the World Health Organization (WHO) introduced the Integrated Care for Older People (ICOPE) guidelines [5], representing a paradigm shift away from a traditional disease-centred model towards a function- and person-centred approach to healthy ageing. The ICOPE guidelines prioritised the optimisation of intrinsic capacity (IC) as a key strategy for fostering healthy ageing. IC is the composite of an individual’s physical and mental capacities (as opposed to deficits), and in turn determines a person’s functional ability after interactions with environmental factors. The WHO operationalised IC into six core domains—locomotion, cognition, psychological capacity, vitality, hearing, and vision—providing a pragmatic framework for assessment, monitoring, and intervention across clinical and community settings [6,7,8].

Importantly, IC is a dynamic construct that evolves across the life course and is potentially modifiable [7,9]. Longitudinal monitoring of IC trajectories enables early identification of declines, facilitates risk stratification, and supports timely preventive or restorative interventions at both individual and population levels. Emerging evidence suggests that declines in IC often precede overt disability and frailty [10], positioning IC as a valuable upstream target for healthy ageing strategies [7]. Lifestyle factors represent a key modifiable determinant of IC [11,12] and are increasingly recognised as central to strategies aimed at extending healthspan. Healthy lifestyle behaviours—including balanced nutrition, regular physical activity, social engagement, and avoidance of harmful behaviours such as smoking—have been shown to correlate positively with multiple IC domains [13,14].

Singapore’s rapidly ageing population is projected to reach super-aged status by 2026, with older adults aged 65 years and above accounting for approximately 20% of residents. Against this demographic backdrop, identifying effective strategies to improve population-level healthspan is of critical importance. While clear associations between lifestyle factors and health outcomes have been established, the combined association of lifestyle behaviours with IC remains relatively understudied [12]. Our study aimed to examine the association between a Healthy Lifestyle Score (HLS) [14] and intrinsic capacity in older adults in a population-based sample of older adults from a health precinct [15] in Singapore.

## 2. Methods

### Study Cohort and Recruitment

The Baseline Study for the Health District @ Queenstown recruited a community-based, multi-ethnic cohort of residents from the Queenstown district in Singapore [16]. To obtain a representative sample, the Department of Statistics (DOS) Singapore provided selected postal codes and addresses stratified by age, race, and housing type. Invitation letters were mailed to these households, supplemented by banners and posters displayed in public areas. Trained interviewers subsequently visited the addresses to invite eligible residents to complete a questionnaire and undergo physical health measurements. Participation was limited to Singapore citizens and permanent residents aged 21 years and above, with up to two individuals per household enrolled. Data collection took place between September 2023 and May 2024, with written informed consent obtained from all participants. The study protocol was approved by the Institutional Review Board of the National University of Singapore (NUS-IRB-2023-297).

Data from older adults aged ≥ 60 years was analysed. Data collected included age, body mass index (BMI), gender, racial identity, level of education, smoking and alcohol status. A modified version of the WHO Integrated Care for Older People (ICOPE) Step 1 screening tool [16] was used to assess intrinsic capacity (IC) which covered 5 domains: cognition, vitality, locomotion, sensory (vision and hearing), and psychological well-being.

The HLS was adapted from prior epidemiological studies [14,17], which consists of five behaviours. These include absence of smoking (defined as never smoked or quit smoking), absence of significant alcohol consumption (defined as less than once per week in the past year), presence of adequate physical activity (defined as a rapid assessment of physical activity [18] aerobic score ≥ 5, corresponding to meeting recommended moderate or vigorous activity levels), adequate sleep duration (defined as at least 7 h of sleep per night), healthy BMI (defined as a BMI range between 18.5 kg/m^2^ and 23 kg/m^2^) [19]. Equal weighting of components is consistent with epidemiological frameworks that conceptualise lifestyle behaviours as modifiable factors exerting cumulative rather than hierarchical effects on health outcomes [14,17], where the combined burden of unhealthy behaviours is emphasised rather than the relative magnitude of any single factor. Each component contributed one point if present, yielding a composite HLS ranging from 0 to 5. Based on the total score, participants were categorised into three lifestyle groups: unhealthy (scores 0–2), intermediate (score 3), and healthy (scores 4–5).

## 3. Statistical Analysis

Descriptive statistics were used to summarise participant characteristics, with continuous variables presented as means and standard deviations (SD), and categorical variables as frequencies and percentages. Between-group comparisons for participants with and without intrinsic capacity (IC) deficits were performed using *t*-tests for continuous variables and chi-square tests for categorical variables.

The HLS was analysed as a categorical variable (unhealthy: 0–2; intermediate: 3; healthy: 4–5). Multivariable logistic regression was performed to examine associations between healthy lifestyle factors and the presence of ≥1 intrinsic capacity (IC) deficit (binary outcome: ≥1 deficit vs. none) [16]. Variables found to be statistically significant in univariate analyses (*p* < 0.05) were considered for inclusion in the multivariable model. In addition, age, sex, BMI (Asian cut-offs), education level, and race were included a priori based on their established relevance to IC and health behaviours, regardless of univariate significance. Lifestyle variables (physical activity, sleep, smoking, and alcohol intake) were entered simultaneously to evaluate their independent associations with IC deficits. Adjusted odds ratios (ORs) with 95% confidence intervals (CIs) were reported. Multicollinearity was assessed using variance inflation factors (VIF), and model fit was evaluated using the Hosmer–Lemeshow goodness-of-fit test. All statistical tests were two-tailed, with statistical significance set at *p* < 0.05. Statistical analyses were performed using IBM SPSS Statistics for Windows, Version 31.0 (IBM Corp., Armonk, NY, USA).

## 4. Results

A total of 1644 participants were analysed, and the results are summarised in Table 1. Participants with IC deficits were significantly older than those without IC deficits (74.5 ± 8.2 vs. 69.6 ± 6.9 years; *p* < 0.001). A slight female preponderance was observed in both groups, with women comprising 54.8% of participants without IC deficits and 56.5% of those with IC deficits, although this was not statistically significant. Mean BMI was similar between groups (23.8 ± 8.3 vs. 23.6 ± 4.6 kg/m^2^; *p* = 0.663).

**Figure 1:**
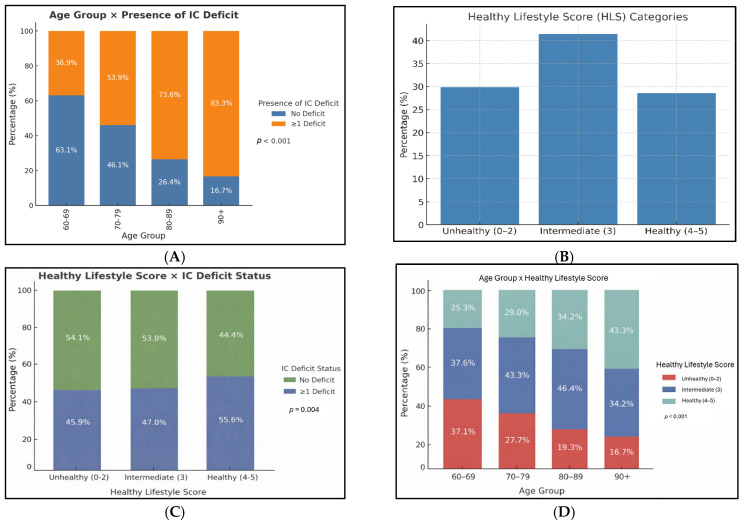
(**A**): Proportion of older adults with ≥1 intrinsic capacity deficit by age group. (**B**): Distribution of Healthy Lifestyle Score categories. (**C**): Association between Healthy Lifestyle Scores and intrinsic capacity deficit status. (**D**): Distribution of Healthy Lifestyle Scores across age groups.

Stratified by age group, the prevalence of intrinsic capacity (IC) deficits increased progressively with advancing age (Figure 1A), demonstrating a statistically significant age-related trend (*p* < 0.001). Among participants aged 60–69 years, 36.9% had at least one IC deficit. This proportion increased to 53.9% in those aged 70–79 years, 73.6% among those aged 80–89 years, and 83.3% in participants aged 90 years and above. Overall, 29.9% of participants were classified as having an unhealthy lifestyle profile, 41.4% had an intermediate lifestyle profile, and 28.6% had a healthy lifestyle profile (Figure 1B). HLS categories were significantly associated with IC deficit status (Figure 1C). Participants with poorer lifestyle profiles had a higher prevalence of IC deficits compared with those with healthier lifestyle profiles (*p* = 0.004). Specifically, 54.1% of participants with an unhealthy lifestyle profile (HLS 0–2) had at least one IC deficit, compared with 44.4% of participants with a healthy lifestyle profile (HLS 4–5).

HLS profiles also varied significantly across age groups (Figure 1D). Among participants aged 60–69 years, 37.1% had an unhealthy lifestyle profile, 37.6% had an intermediate profile, and 25.3% had a healthy profile. In the 70–79-year age group, these proportions were 27.7%, 43.3%, and 29.0%, respectively. Among those aged 80–89 years, 19.3% had an unhealthy profile, 46.4% had an intermediate profile, and 34.2% had a healthy profile, while in participants aged 90 years and above, 16.7% had an unhealthy profile, 40.0% had an intermediate profile, and 43.3% had a healthy profile. Overall, there was a statistically significant trend toward a higher proportion of healthier lifestyle profiles with increasing age (*p* < 0.001).

The associations between intrinsic capacity (IC) deficits and individual risk factors were examined using multivariable logistic regression models (Table 2). Increasing age was independently associated with higher odds of IC deficits (OR 1.08 per year, 95% CI 1.07–1.10; *p* < 0.001). Insufficient physical activity was strongly associated with IC deficits (OR 2.28, 95% CI 1.66–3.15; *p* < 0.001), as was insufficient sleep (OR 1.89, 95% CI 1.50–2.38; *p* < 0.001). Race was also independently associated with IC deficits (OR 1.28, 95% CI 1.06–1.56; *p* = 0.012). Alcohol intake (at least once per week in the past year) was inversely associated with IC deficits (OR 0.66, 95% CI 0.50–0.88; *p* = 0.004). Sex (OR 1.06, 95% CI 0.84–1.33; *p* = 0.645), BMI based on Asian cut-offs (OR 1.14, 95% CI 0.92–1.42; *p* = 0.217), education level (OR 0.93, 95% CI 0.80–1.07; *p* = 0.315), and smoking (OR 1.02, 95% CI 0.68–1.52; *p* = 0.938) were not significantly associated with IC deficits in the adjusted models.

## 5. Discussion

This study examined the association between intrinsic capacity deficits and the HLS among community-dwelling older adults. A high prevalence of intrinsic capacity deficits was observed, particularly at older ages, and only 28% of participants were categorised as healthy by their HLS. Compared with participants with unhealthy or intermediate HLS, those with a healthy HLS had a lower prevalence of at least one intrinsic capacity deficit. This indicates a positive association between healthier lifestyle behaviours, as reflected by HLS, and intrinsic capacity. These findings are consistent with prior studies from China and India, which similarly reported higher intrinsic capacity or lower IC impairment among older adults with healthier lifestyle behaviours, alongside a higher burden of IC deficits at older ages [14,20,21].

In terms of specific lifestyle behaviours, insufficient physical activity showed the strongest association with intrinsic capacity deficits, with a more than two-fold increase in odds. Increased physical activity can lead to lower incidence of motor disability, improved mood, cognition, and quality of life. Physical activity is a key determinant of muscle health and a central modifiable factor in the prevention of sarcopenia [22]. Regular physical activity supports multiple domains of intrinsic capacity, including mobility, vitality, cognitive function, and psychological well-being [23]. By attenuating age-related declines in muscle strength, balance, and cardiometabolic health [24], physical activity can reduce intrinsic capacity deficits that increase vulnerability to frailty, disability, and loss of independence. At a population level, sustained engagement in physical activity contributes to healthier ageing trajectories, enhanced functional resilience, and improvements in healthspan within ageing communities [16].

Insufficient sleep showed the second strongest association with intrinsic capacity (IC) deficits, corresponding to approximately 1.8-fold higher odds. Poor sleep and impaired circadian rhythms are postulated to be associated with chronic inflammatory states, leading to increased risks of hypertension, cardiovascular diseases, and mortality [25]. In the HLS, sufficient sleep was defined as at least 7 h of sleep per night. A study by Quan et al. [26] showed that at least 7.5 h of sleep is needed to avoid a decrease in intrinsic capacity, and that good sleep quality is also important, in addition to merely sleep duration. Depressed mood can also lead to disturbances in the circadian rhythm and is closely linked with poor sleep [26], and negative sleep patterns are also associated with impaired cognitive function [27]. Of the five intrinsic capacity domains, at least two—psychological and cognitive capacity—are closely related to sleep. Longitudinal studies are therefore warranted to examine whether improvements in sleep health—encompassing quality, duration, regularity, and sleep efficiency—translate into sustained gains in intrinsic capacity over time. In parallel, community-based efforts to promote non-pharmacological sleep hygiene practices, coupled with early screening for cognitive and mood disturbances, may facilitate earlier intervention and support the preservation of intrinsic capacity.

Both intact vision and being unemployed or retired were significantly associated with lower odds of unhealthy lifestyle behaviours. Preserved visual function may facilitate greater social participation, enhance physical functioning, and broaden life-space mobility among older adults [28], which could be vital in preserving functional capacity. Similarly, those who are unemployed or retired may have more temporal flexibility to participate in health-promoting lifestyle behaviours. Males had a significantly higher odds ratio of having an unhealthy lifestyle score. This finding is consistent with prior epidemiological evidence indicating that men, across diverse settings, are more likely to engage in certain health-risk behaviours, including tobacco use, alcohol consumption, and lower uptake of preventive health practices [29,30]. Differences in occupational patterns, social norms, and stress exposures in highly urbanised environments such as Singapore may further shape these lifestyle behaviours, although causal pathways cannot be inferred from the present analysis.

Our study has several limitations. First, the cross-sectional design precludes inference of causal relationships between the HLS and IC. As IC is a dynamic construct that evolves across the life course, a single time-point assessment cannot capture trajectories or within-person changes over time. Reverse causation is also plausible; individuals with IC deficits may modify their behaviours (e.g., reduce physical activity or alcohol consumption) in response to declining health. Accordingly, the observed associations should be interpreted as correlational rather than causal. Longitudinal studies are warranted to clarify temporal relationships and to determine whether sustained healthy lifestyle behaviours influence IC trajectories. Second, the HLS relied predominantly on self-reported measures, introducing the potential for recall and reporting bias. Third, the assessment did not differentiate between levels or patterns of alcohol consumption, nor did it incorporate objective measures of sleep quality or physical activity intensity, which may have limited the granularity of exposure assessment. Lastly, our dataset did not include detailed measures of social participation, loneliness, caregiving burden, or living arrangements. The absence of these variables limits our ability to account for potentially important social determinants that may influence the observed associations.

## 6. Conclusions

Healthier lifestyle profiles are significantly associated with fewer IC deficits. These findings underscore the importance of targeting modifiable health behaviours to preserve intrinsic capacity and support healthy ageing. In particular, physical activity, sleep, and vision are important targets to address IC deficits amongst older adults. Longitudinal follow-up studies are needed to establish temporal and potentially causal relationships between healthy lifestyle profiles and IC deficits.

## Figures and Tables

**Table 1:** Baseline characteristics of participants with and without intrinsic capacity (IC) deficits.

Variable	*Without IC Deficit (n, %, Mean ± SD)*	*With IC Deficit (n, %, Mean ± SD)*	*p-Value*
Age (years)	69.6 ± 6.9 (n = 808)	74.5 ± 8.2 (n = 836)	<0.001
BMI (kg/m^2^)	23.8 ± 8.3 (n = 808)	23.6 ± 4.6 (n = 836)	0.663
Sex (Female)	443 (54.8%)	472 (56.5%)	0.505
Race			0.056
Chinese	742 (91.8%)	742 (88.8%)	
Malay	29 (3.6%)	33 (3.9%)	
Indian	28 (3.5%)	53 (6.3%)	
Others	9 (1.1%)	8 (1.0%)	
Education			<0.001
Primary	488 (60.4%)	601 (71.9%)	
Secondary	136 (16.8%)	119 (14.2%)	
Tertiary & above	184 (22.8%)	116 (13.9%)	
Smoking (Yes)	70 (8.7%)	63 (7.5%)	0.402
Alcohol (Yes)	206 (25.5%)	122 (14.6%)	<0.001

**Table 2:** Multivariate logistic regression of healthy lifestyle factors with IC deficits *.

Variable	OR (95% CI)	*p* Value
Age	1.08 (1.07–1.10)	<0.001
Male	1.06 (0.84–1.33)	0.645
Suboptimal BMI (Asian cut-offs)	1.14 (0.92–1.42)	0.217
Physical activity (Insufficient)	2.28 (1.66–3.15)	<0.001
Sleep (Insufficient)	1.89 (1.50–2.38)	<0.001
Smoking	1.02 (0.68–1.52)	0.938
Alcohol intake	0.66 (0.50–0.88)	0.004

* Analysis adjusted for age, sex, race, educational level and BMI (Asian cut-offs). Lifestyle variables (physical activity, sleep, smoking, and alcohol intake) were entered simultaneously. OR = odds ratio; CI = confidence interval. Model diagnostics: all variance inflation factors (VIF) < 1.2, indicating no multicollinearity; Hosmer–Lemeshow goodness-of-fit test χ^2^ = 3.60, df = 8, *p* = 0.891, indicating good model fit.

## Data Availability

The data presented in this study are available on reasonable request from the corresponding author. The data are not publicly available due to privacy and ethical restrictions.
